# Perioperative Hemodynamic Optimization in Patients at Risk for Delirium – A Randomized-Controlled Trial

**DOI:** 10.3389/fmed.2022.893459

**Published:** 2022-07-13

**Authors:** Kristina E. Fuest, Ariane Servatius, Bernhard Ulm, Stefan J. Schaller, Bettina Jungwirth, Manfred Blobner, Sebastian Schmid

**Affiliations:** ^1^Department of Anesthesiology and Intensive Care, Technical University of Munich, School of Medicine, Klinikum rechts der Isar, Munich, Germany; ^2^Department of Anesthesiology and Operative Intensive Care Medicine, Charité – Universitätsmedizin Berlin, Corporate Member of Freie Universität Berlin, Humboldt-Universität zu Berlin and Berlin Institute of Health, Berlin, Germany; ^3^Department of Anesthesiology and Intensive Care Medicine, University Hospital Ulm, Ulm University, Ulm, Germany

**Keywords:** outcome, post-operative delirium, goal-directed hemodynamic monitoring, goal-directed therapy, frailty

## Abstract

**Background:**

Post-operative delirium is common in elderly patients and associated with increased morbidity and mortality. We evaluated in this pilot study whether a perioperative goal-directed hemodynamic optimization algorithm improves cerebral oxygenation and can reduce the incidence of delirium.

**Materials and Methods:**

Patients older than 70 years with high risk for post-operative delirium undergoing elective non-cardiac surgery were randomized to an intervention or control group. Patients in the intervention group received a perioperative hemodynamic optimization protocol based on uncalibrated pulse-contour analysis. Patients in the control group were managed according to usual standard of care. Incidence of delirium until day seven was assessed with confusion assessment method (CAM) and chart review. Cerebral oxygenation was measured with near-infrared spectroscopy.

**Results:**

Delirium was present in 13 of 85 (15%) patients in the intervention group and 18 of 87 (21%) in the control group [risk difference −5.4%; 95% confidence interval, −16.8 to 6.1%; *P* = 0.47]. Intervention did not influence length of stay in hospital or in-hospital mortality. Amounts of fluids and vasopressors applied, mean arterial pressure, cardiac index, and near-infrared spectroscopy values were comparable between groups.

**Conclusion:**

The hemodynamic algorithm applied in high-risk non-cardiac surgery patients did not change hemodynamic interventions, did not improve patient hemodynamics, and failed to increase cerebral oxygenation. An effect on the incidence of post-operative delirium could not be observed.

**Clinical Trial Registration:**

[Clinicaltrials.gov], identifier [NCT01827501].

## Introduction

Delirium is a common post-operative complication in the elderly (1). It is defined as an acute neuropsychiatric disorder characterized by fluctuations in attention, awareness, and cognition. The incidence depends on several factors like age, number of comorbidities, pre-operative cognitive or functional impairment, and type of surgery (2–4). Particularly in patients admitted to the intensive care unit (ICU) the incidence is between 50 and 80% (5) and their length of stay in ICU and hospital is prolonged. As a consequence, delirium is both a huge burden on a patient’s wellbeing and on the healthcare system overall (6, 7). To prevent delirium multimodal and multidisciplinary interventions should be implemented during hospital stay and particularly in the perioperative course (8).

Since maintaining a sufficient perfusion is a general principle in anesthesia, the patient’s hemodynamic status could be one target point of intervention. Sufficient perfusion and oxygen delivery are essential in order to avoid impairment of the brain (9). In sepsis-associated delirium a correlation with cerebral perfusion pressure has been demonstrated as one of many contributing factors (10). In addition, the correlation between intraoperative hypotension and post-operative delirium has been shown in a recent clinical trial not exclusively for sepsis but also involving surgical patients (11). Furthermore, poorer cerebral perfusion pressure was associated with a higher risk of post-operative delirium as well as longer duration and higher severity of delirium, independent of demographic and medical predictors in a cohort of lung-transplant recipients (12). This indicates that individual adjustment of cerebral perfusion in terms of goal-directed hemodynamic optimization could be an approach to reduce the incidence of delirium *via* improvement of cerebral oxygenation especially in elderly patients (13). Uncalibrated pulse contour analysis requires only an arterial line and cardiac index is calculated with an algorithm using bodyweight and height of the patient. It allows efficient, continuous monitoring, targeting of optimal cardiac output and facilitates management of vasopressors and fluid administration during high-risk surgery (14, 15). As a consequence, intraoperative cerebral perfusion and oxygenation might be optimized by being able to target cardiac output measures. The hypothesis of our study is, that goal-directed hemodynamic optimization will improve cerebral perfusion and consequently cerebral oxygenation and thereby reduce the incidence of delirium in a high-risk population compared with standard therapy.

## Methods

We performed a prospective, randomized, single-center study at a university hospital in Munich, Germany. Patients older than 70 years with a high risk of developing post-operative delirium were included and randomized into two treatments arms: intervention and control group.

The study was approved by the ethics committee of the Technical University of Munich (Ethikkommission der Technischen Universität München, Ismaninger Straße 22, 81675 München; Approval Number: 5687/13 S on February 28th, 2013 and October 24th, 2018; Chairperson Prof. Dr. G. Schmidt) and prospectively registered at Clinical Trials (April 2013; NCT01827501). There was one amendment to the study in 2018, when a new German law for data protection regulation has come into force and the patient information sheet had to be updated. The study was conducted in accordance with the Declaration of Helsinki.

### Eligibility Criteria and Randomization

Patients were screened for eligibility during the pre-anesthesia visit. Inclusion criteria were age above 70 years, major elective non-cardiac surgery (defined by a scheduled surgery time ≥90 min) and a high risk for delirium (screening score ≥6 points; see below). Surgical procedures included all types of surgery except cardiac, major aortic, and neurosurgery. Patients with emergency procedures and patients who had general anesthesia within the last 30 days were excluded. Further exclusion criteria were valvular disorders grade II or higher as well as history of major aortic surgery, as these factors are known to distort the uncalibrated pulse contour analysis. A detailed interview with the patient and/or his caregivers was conducted by a research team member during the pre-anesthesia visit to assess the patient’s risk for delirium. Several predisposing and precipitating factors were identified and scored with one or two points according to Table 1. The scoring system is a modification of the risk score described by Marcantonio based on the work of Inouye (16, 17). We included only patients with a score of ≥6 points in the study as these patients have a high risk of at least 30% for delirium (18).

**TABLE 1:** Screening score: risk factors for delirium.

Risk factor category	Predisposing factors	Precipitating factors
Major (2 points)	• Advanced age (≥80 years)	• High-risk surgical procedure*
	• Dementia (MMSE <20) or recent delirium, not resolved	• Planned intensive care unit stay ≥2 days
Minor (1 point)	• Older age (70–79 years)	• Moderate-risk surgical procedure*
	• Mild cognitive impairment (MMSE ≤24)	• General anesthesia
	• History of stroke	• Planned intensive care unit stay <2 days
	• Functional disability (MET <4, paresis, hearing aid, glasses)	
	• Laboratory abnormalities	
	• High medical comorbidity, including cardiovascular risk factors	
	• Alcohol/sedative abuse	
	• Depressive symptoms	

*High-risk surgical procedures include open vascular and major abdominal surgery.

*Moderate-risk surgical procedure include orthopaedic, ear, nose and throat, gynaecologic and urologic surgery.

*MMSE, Mini-Mental-State Examination; MET, Metabolic Equivalent of Task.*

A research team member evaluated the patients’ eligibility, informed the patient in detail about the study, and obtained written informed consent. He enrolled the patient and assigned him to intervention or control group in a 1:1 ratio. The randomization list was generated by a study team member using a random generator without blocks (Microsoft Excel for Mac 14.0). For each randomization number we prepared a paper-based folder with all required materials including the group assignment. Only the folder with the lowest number was accessible to the study team member responsible for the allocation.

### Pre-operative Predisposing Factors

A Mini-Mental-State exam (MMSE) was performed to detect dementia or cognitive impairment (MMSE ≤ 24: mild cognitive impairment; MMSE > 20 and < 24: dementia) and the Confusion assessment method (CAM-) Score was obtained (19, 20). Patients with present delirium were excluded. Above that, activities of daily life were assessed to determine the presence of frailty according to the Clinical Frailty Scale (CFS) (21). As already determined in large multicentric international trials, patients with a CFS 5–8 were considered as frail (22). Above that, functional disability is present, when sensory or visionary aids are necessary, walking sticks, rollators or wheelchairs are required or patients need feeding, e.g., by a percutaneous endoscopic gastrostomy. To determine high medical comorbidities and cardiovascular risk factors medical records including clinical charts and nursing records were reviewed. Data collection included patient biometrics, comorbidities, clinical parameters and laboratory findings. The risk of the surgical-procedures was determined according to the German Society of Anesthesia (23).

### Perioperative Treatment

After transfer to the operating theater, an arterial line was introduced *via* Seldinger technique in the radial (3 French) or femoral (4 French) artery under local anesthesia before induction of anesthesia. Following the induction of general anesthesia with sufentanil, propofol and rocuronium the patient was intubated. Anesthesia was maintained with sufentanil, rocuronium and sevoflurane. Depth of anesthesia was recorded using entropy and was kept between 40 and 60. A central venous catheter was placed when necessary, according to the attending specialist.

**FIGURE 1:**
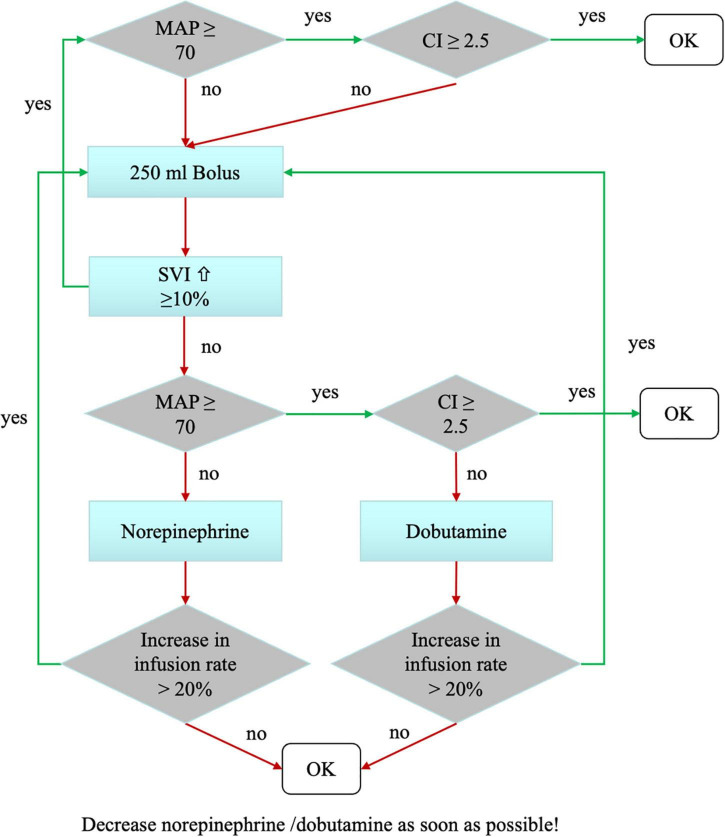
Hemodynamic treatment algorithm in the intervention group. The hemodynamic goals were mean arterial pressure (MAP) >70 mmHg and a cardiac index (CI) >2.5 L/kg/m^2^. Hemodynamics were evaluated routinely every 30 min, as well as at times of hemodynamic instability. We tested fluid responsiveness using a volume challenge of 250 mL Ringer’s acetate. Depending on changes in the stroke volume index (SVI), the patient received either volume or catecholamines in a titrated manner.

In the goal-directed hemodynamic optimization group (group intervention) hemodynamic management was performed according to a previously published algorithm obtained by pulse contour analysis using the PulsioFlex^®^ device (PULSION Medical Systems SE; Feldkirchen; Germany) (see Figure 1) (24). Evaluation of the algorithm was started before induction of anesthesia and continued until discharge from the post-anesthesia care unit (PACU).

For volume therapy Ringer’s acetate was used. Every patient received a basal infusion with a dosage of 1 ml/kg per hour according to our standard care. The algorithm was based on the two factors mean arterial blood pressure and cardiac index. If both factors were in a sufficient range (MAP > 70 mmHg, cardiac index > 2.5 L/kg/m^2^), no intervention was necessary. In case of insufficient mean arterial pressure or cardiac index, the patient received a fluid bolus of 250 ml Ringer’s acetate in 5–10 min followed by another assessment of the algorithm. If after a fluid bolus the stroke volume index did not increase, drug therapy was initiated. Norepinephrine was used as vasopressor, Dobutamine as inotropic medication.

The goal-directed optimization was terminated, when the patient fulfilled the standard criteria for discharge from recovery room: the pain level was ≤3 according to the numeric rating scale (NRS), the hemodynamic situation was stable without catecholamines, the pulmonary situation was stable, and the patient fully awake and compliant.

In the control group an arterial line was placed as well and hemodynamic management was performed according to heart rate and blood pressure without using extended monitoring like pulse contour analysis or any other goal-directed hemodynamic monitoring. Ringer’s acetate was used for fluid replacement at the attending anesthetist’s discretion. Here, the responsible anesthetist was blinded to the results of the goal-directed hemodynamic monitoring which was obscured by the study team.

In both groups other medication like antibiotics, anticoagulants, or pain medication was administered according to the intraoperative standard operating procedure protocol of the department of anesthesia and intensive care medicine. Patients in all groups received red blood cell transfusion, when hemoglobin value dropped below 8 mg/dl or in case of cardiac impairment such as ST-depression. Coagulation factors and fresh frozen plasma were substituted according to the coagulation status assessed with rotational thromboelastometry (ROTEM™).

### Near-Infrared Spectroscopy

To test our hypothesis, that goal-directed hemodynamic optimization reduces delirium by improving perfusion and consequently enhancing oxygenation, it was necessary to measure the oxygen concentration of the tissue. In the last years NIRS has been introduced in daily clinical practice (25). The device is safe, non-invasive and was used in our patients to assess the oxygen concentration in the brain. In this study, the INVOS™ (Medtronic GmbH, Earl-Bakken-Platz 1, 40670 Meerbusch, Germany) cerebral somatic oximeter with two adult sensors placed on the left and right side of the forehead was used.

To evaluate the difference to pre-operative values, the monitoring was established before induction of anesthesia in both groups. In the intervention group values from NIRS monitoring were available to the responsible anesthetist. However, they were not included in the hemodynamic optimization algorithm. Thus, it was left to the treating anesthesiologist to react individually to possible insufficient NIRS values in the intervention group. Anesthesiologists in the control group were blinded to the NIRS monitoring.

### Data Collection

Following data were recorded: demographics (age, sex, and co-morbidities) and information obtained during the pre-anesthesia visit including predisposing factors for delirium, surgical and anesthesiologic data extracted from the anesthesia and surgical protocol (including medication and administered fluids, goal-directed hemodynamic monitoring parameters as well as NIRS and entropy), parameters obtained during the post-operative visit that can be extracted from the clinical charts on the ward, length of stay in hospital, and follow-up data like mortality after 1 year.

### Detection of Delirium, Post-operative Visit and Follow-Up

For detection of delirium the CAM Score (19, 26, 27) was obtained from every patient once daily until day 7 after surgery. This was done by a member of the study team who had been thoroughly trained. If delirium was present, the severity was also assessed using CAM-S (28, 29). Delirium often occurs during the night. Since the visit by the study team took place during the day, we decided to improve detection of delirium by inspecting the patient files to review delirium associated medication like haloperidol and by exchanging information with the ward team. This ensured that even in the event of a poor handover of the night shift to the day shift, abnormalities during the night became apparent and could thus be evaluated with the treating team if necessary. Furthermore, inadequate qualification of the nursing staff (such as inexperienced in delirium symptoms and their clinical presentation) could thus be compensated for *via* the evaluation of the patient record. As delirium can be triggered by pain the NRS was registered daily. If the patient was admitted to ICU, the CAM was also obtained there. As in anesthetized and ventilated patients obtaining the CAM is not possible, we performed an additional sensitivity analysis and assigned these patients to the delirium group assuming worst outcome (worst-case imputation). Furthermore, a second MMS-Test was performed in every patient on day 7 or the day before discharge, whichever occurs first. Patients were subsequently followed for up to 1 year after surgery *via* telephone interview to assess mortality. In cases where we were not able to reach the patient, we reviewed the hospital record for information about survival during the last year.

### Primary and Secondary Endpoints

Primary endpoint was the incidence of delirium until day 7. Duration of delirium as well as the day it first occurred were investigated as secondary endpoints. Further secondary endpoints were: length of stay in hospital, in-hospital mortality, and mortality after 1-year.

### Blinding

Patients were blinded to group allocation and intervention throughout the trial. Anesthesiologists treating the patient during surgery and in the PACU were not blinded but in the control group they were not able to assess the parameters of the goal-directed hemodynamic and NIRS monitoring. Nurses and physicians treating the patient on ICU or normal ward after surgery were blinded to group allocation. The outcome assessor was not blinded to the intervention.

### Statistical Analyses

Data analysis was performed with R version 4.1.0. Continuous variables are presented as median [interquartile range (IQR)]. Categorical variables are presented using absolute numbers and frequencies. Effect sizes were calculated using differences in median for continuous variables and risk difference (RD) for binary variables. In addition to effect sizes null hypothesis tests were conducted *via* Mann-Whitney *U*-tests for continuous variables and by χ^2^–tests for binary variables. To validate our results, we performed a sensitivity analysis using worst-case imputation. A two-sided *P*-value of less than 0.048 was considered statistically significant.

### Sample Size Calculation

By using a screening score we intended to included patients with an expected delirium-incidence above 30%. As in most interventional studies the risk for post-operative delirium could be reduced by one third these figures could have been used for sample size calculation (4, 6). However, 6 or more points in the screening score correspond to a wide range of delirium-incidence between 30 and 50%. As the actual incidence had a significant impact on the number needed per group, we *a priori* planned an interim analysis after 100 included patients to assess the new sample size according to a modification of the O’Brian-Fleming technique (30). In the interim analysis the incidence of delirium was 3/47 (6%) in the intervention and 11/52 (21%) in the control group (*P* = 0.04; Fisher’s exact test; 1 patient excluded as pre-set surgery time was not adhered). As the difference of delirium between the two groups was not significant with a pre-defined α < 0.002 to finish the study, the sample size needed per group was adjusted. Thereafter, based on two-tailed χ^2^-test, assuming an α = 0.048 and a power of 80%, the analysis disclosed 86 patients per group. The calculation was performed *via* DataTab (URL).^1^ As a result, of the *a priori* planned interim analysis, the targeted sample size was now set to 172.

## Results

**FIGURE 2:**
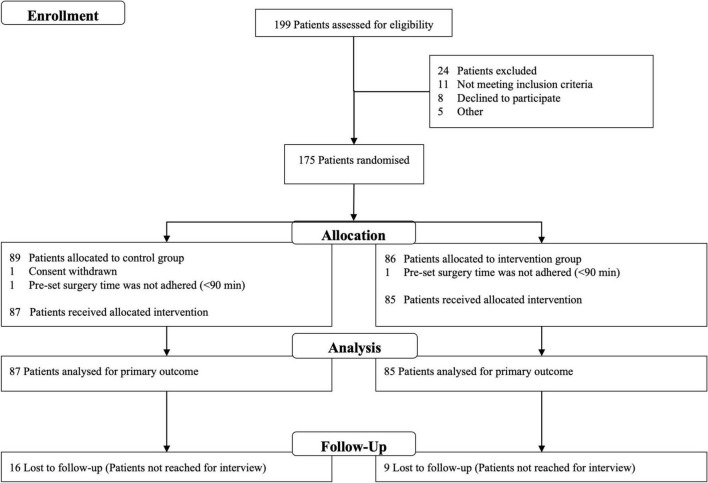
CONSORT diagram.

Between May 2013 and December 2019, 172 patients were included in the study. Follow-up was finished in February 2021. Figure 2 shows the CONSORT diagram of the study. Surgical procedures included all departments with abdominal surgery being the most frequent. 85 patients were randomized into the intervention group. 87 patients received standard care. Baseline characteristics were comparable between the two groups regarding screening score, frailty, and pre-medical condition (Table 2).

**TABLE 2:** Characteristics after randomization into the two groups.

	Control *N* = 87	Intervention *N* = 85
Age (yr), median [IQR]	79 [74 to 82]	77 [74 to 82]
Female, n/total N (%))	37 (43%)	36 (42%)
BMI, median [IQR]	24.6 [23.0 to 28.7]	24.7 [22.5 to 27.4]
Delirium risk score, median [IQR]	7 [6 to 8]	7 [7 to 8]
Delirium risk score components, n/total N (%)		
6	28 (32%)	19 (23%)
7	28 (32%)	35 (41%)
8	20 (23%)	22 (26%)
9	7 (8%)	7 (8%)
10	4 (5%)	2 (2%)
ASA, median [IQR]	3 [2 to 3]	3 [2 to 3]
ASA III, n/total N (%)	57 (66%)	63 (74%)
Clinical frailty scale, median [IQR]	4 [3 to 4]	4 [3 to 5]
Clinical frailty scale components, n/total N (%)		
1-4	67 (77%)	61 (72%)
5-9	20 (23%)	24 (28%)
Preoperative MMSE, median [IQR]	27 [25 to 29]	28 [26 to 29]
Preoperative MMSE components, n/total N (%)		
Dementia	1 (1%)	3 (3%)
Cognitive impairment	21 (24%)	15 (18%)
Unobtrusive	65 (75%)	67 (79%)
Surgical department, n/total N (%)		
Abdominal surgery	66 (76%)	54 (64%)
Vascular surgery	5 (6%)	14 (16%)
Orthopedics	12 (14%)	13 (15%)
Trauma surgery	4 (4%)	4 (5%)
Type of surgery, n/total N (%)		
High-risk surgery (open vascular abdominal, oesophageal)	7 (8%)	6 (7%)
Moderate-risk Surgery (abdominal, orthopedic)	74 (85%)	75 (88%)
Anesthesia time (min), median [IQR]	274 [203 to 364]	278 [218 to 407]
Surgical time (min), median [IQR]	190 [115 to 260]	180 [130 to 300]
Comorbidities, n/total N (%)		
Diabetes mellitus	24 (28%)	21 (25%)
Arterial hypertension	70 (81%)	66 (78%)
Cardiac risk factors, n/total N (%)	65 (75%)	71 (84%)
Coronary artery disease	39 (45%)	38 (45%)
Heart failure	20 (23%)	21 (25%)
Arrhythmia	13 (15%)	32 (38%)
History of stroke, n/total N (%)	7 (8%)	10 (12%)
History of delirium, n/total N (%)	2 (2%)	2 (2%)

*BMI, Body-Mass-Index; ASA, American Society of Anesthesiologists; MMSE, Mini-Mental-State Examination; IQR, interquartile range.*

### Components of the Goal-Directed Therapy and Cerebral Oxygenation

The number of crystalloids, colloids, blood products, and vasopressors infused was comparable between groups. Patients in the intervention group received more inotropes. Regarding hemodynamics patients in the control group had an increased MAP, whereas cardiac index was higher in the intervention group (Table 3). NIRS monitoring showed comparable cerebral oxygenation (median and delta from pre-induction) in both groups during surgery [median NIRS total: control 68 (IQR 78 to 96) vs. intervention 81 (75 to 88); median difference 2.8; 95% confidence interval (CI) −0.9 to 6.0; *P* = 0.09; Table 3] and in the PACU (Table 3).

**TABLE 3:** Comparison of perioperative parameters.

	Control *N* = 87	Intervention *N* = 85	Effect size [95% CI]	*P*-value
Fluids and catecholamines during surgery				
Ringer’s acetate (ml), median [IQR]	2400 [1850 to 3400]	3100 [1900 to 4500]	-700 [-1050 to 300]	0.08
Gelatine administered, n/total N (%)	4 (5%)	3 (4%)	-1.1% [-7 to 4.8%]	1.00
Albumin administered, n/total N (%)	13 (15%)	18 (21%)	6.2% [-5.2 to 17.7%]	0.39
Blood products administered, n/total N (%)	5 (6%)	12 (14%)	8.4% [-0.5 to 17.2%]	0.11
Mean inotropic (μg kg^–1^ min^–1^), median [IQR]	0 [0 to 0]	0 [0 to 1.8]	0 [0 to 0]	<0.001
Mean vasopressors (μg kg^–1^ min^–1^), median [IQR]	0.04 [0.03 to 0.06]	0.04 [0.02 to 0.07]	0 [-0.02 to 0]	0.67
*Fluids in PACU*				
Ringer’s acetate (ml), median [IQR]	1500 [0 to 2700]	1850 [0 to 3200]	-350 [-2058.7 to 925.4]	0.15
Gelatine administered, n/total N (%)	2 (2%)	1 (1%)	-1.1% [-5 to 2.8%]	1.00
Albumin administered, n/total N (%)	10 (12%)	19 (23%)	10.9% [-0.2 to 22%]	0.10
Blood products administered, n/total N (%)	11 (13%)	6 (7%)	-5.6% [-14.4 to 3.3%]	0.32
Hemodynamics during surgery				
MAP (mmHg), median [IQR]	86 [78 to 96]	81 [75 to 88]	5.2 [-2 to 10.1]	0.02
Heartrate (bpm), median [IQR]	60 [54 to 68]	60 [56 to 70]	0.3 [-4.5 to 3.5]	0.34
Cardiac index (l min m^–2^), median [IQR]	2.5 [2.2 to 3.0]	2.7 [2.6 to 3.1]	-0.2 [-0.4 to -0.1]	<0.001
Hemodynamics in PACU				
MAP (mmHg), median [IQR]	85 [77 to 94]	83 [79 to 95]	2.8 [-3.2 to 6.5]	0.86
Heartrate (bpm), median [IQR]	72 [65 to 79]	69 [62 to 80]	2.7 [-2.2 to 7]	0.61
Cardiac Index (l min m^–2^), median [IQR]	3.1 [2.6 to 3.5]	3.3 [2.7 to 3.8]	-0.2 [-0.5 to 0.2]	0.15
NIRS and entropy during surgery				
NIRS total, median [IQR]	68 [63 to 72]	65 [59 to 70]	2.93 [-0.88 to 6.03]	0.09
NIRS left (%), median [IQR]	70 [63 to 74]	66 [60 to 71]	4.4 [-0.5 to 7.1]	0.05
NIRS right (%), median [IQR]	67 [62 to 72]	65 [60 to 70]	1.8 [-0.8 to 5.7]	0.16
Delta NIRS left (pre-induction and mean during surgery) (%), median [IQR]	-4 [-7 to 0]	-4 [-8 to -1]	-0.1 [-4.1 to 3.1]	0.84
Delta NIRS right (pre-induction and mean during surgery) (%), median [IQR]	-3 [-8 to 2]	-4 [-7 to 0]	-1.6 [-5.4 to 2.8]	0.50
SE during surgery, median [IQR]	40 [34 to 47]	42 [33 to 49]	-2 [-5.1 to 3.7]	0.68
NIRS in PACU				
NIRS left (%), median [IQR]	64 [59 to 70]	63 [60 to 69]	1 [-2.8 to 4.2]	0.68
NIRS right (%), median [IQR]	64 [58 to 69]	62 [58 to 68]	2.1 [-2.8 to 5.7]	0.40

*Blood products include red blood cells, thrombocytes and fresh frozen plasma. All blood products including gelatine and albumin are presented as the number of the patients, who received therapy: n/total N (%).*

*CI, confidence interval; IQR, interquartile range; ICU, intensive care unit; MAP, Mean Arterial Pressure; PACU, Post-anaesthesia Care Unit; NIRS, Near-Infrared- Spectroscopy; SE, State Entropy.*

### Primary Outcome

Delirium was present in 13 patients in the intervention group (15%) and in 18 in the control group (21%) (RD, −5%; 95% CI, −16.8 to 6.1%; *P* = 0.47) (Table 4). The type of delirium assessment (CAM score or chart review) had no influence on the results (Table 4). In an additional sensitivity-analysis with anesthetized patients included in the group of delirium (worst-case imputation), there was no difference between groups (23 vs. 18%) (RD, −5%; 95% CI, −17 to 7%; *P* = 0.50). Length of delirium as well as severity of delirium was not significantly different between groups [2 (1 to 3) vs. 1 (1 to 2)], difference in medians, 1; 95% CI 1 to 2; *P* = 0.27).

**TABLE 4:** Primary and secondary endpoints in the two groups.

	Control *N* = 87	Intervention *N* = 85	Effect size [95% CI]	*P*-value
**Primary endpoint**				
Occurrence of delirium, n/total N (%)	18 (21)	13 (15)	-5.4 [-16.8 to 6.1]	0.470
Occurrence of delirium by CAM, n/total N (%)	16 (18)	11 (13)	-5 [-16 to 5]	0.440
Occurrence of delirium by chart review, n/total N (%)	6 (7)	5 (6)	-1 [-8 to 6]	1.000
Occurrence of delirium (worst-case imputation), n/total N (%)	20 (23)	15 (18)	-5 [-17 to 7]	0.496
Length of delirium (days), median [IQR]	2 [1 to 3]	1 [1 to 2]	1 [-1 to 2]	0.265
Severity of Delirium (CAM-S), median [IQR]	0 [0 to 2]	0 [0 to 1]	0 [0 to 0]	0.120
**Secondary endpoints**				
NRS, median [IQR]	2.3 [1.1 to 3.3]	2.0 [0.9 to 3.0]	0.29 [-0.25 to 0.85]	0.173
Δ MMSE, median [IQR]	0 [-1 to 1]	0 [-1 to 1]	0 [-1 to -1]	0.948
Number of patients admitted to ICU, n/total N (%)	48 (55)	51 (61)	4.8 [-9.9 to 19.6]	0.501
LOS ICU (days), median [IQR]	1 [0 to 1]	1 [0 to 1]	0 [-1 to 0]	0.396
LOS Hospital (days), median [IQR]	11 [8 to 17]	10 [7 to 16]	1 [-2 to 4]	0.414
In-hospital mortality, n/total N (%)	6 (7)	3 (4)	-3.3 [-10.0 to 3.2]	0.529
**Long term secondary outcome**				
Lost-follow up, n/total N (%)	16 (18)	9 (11)	-7.8 [-18.2 to 2.6]	
One-year mortality, n/total N (%)	24 (34)	12 (16)	-13.5 [-25.4 to -1.5]	*0.028*

*CAM, Confusion Assessment Method, CAM-S, Severity of the Confusion Assessment Method, NRS, Numeric Rating Scale, MMSE, Mini Mental State Examination, LOS, Length of Stay, ICU, Intensive Care Unit.*

### Secondary Outcomes

There was no significant difference between groups regarding length of stay in hospital as well as in-hospital-mortality (Table 4). One-year mortality was reduced in the intervention group (12 vs. 24%) (RD, −14%; 95% CI, −25.4 to −1.5%; *P* = 0.03) (Table 4).

## Discussion

In this single-center, randomized-controlled pilot trial a goal-directed hemodynamic optimization algorithm did not lead to significantly different therapeutic interventions, and thus did not result in different hemodynamics or values of cerebral oxygenation. Consequently, the algorithm applied did not reduce post-operative delirium in elderly high-risk patients. There was no effect on secondary endpoints like length of stay in hospital as well as in-hospital-mortality. This monocentric trial does not support the use of this goal-directed hemodynamic optimization algorithm in the prevention of post-operative delirium.

The incidence of post-operative delirium with 21% in the control group and 15% in the intervention group (*P* = 0.47) was low compared to an anticipated occurrence of delirium of a least 30% in our high-risk population (3). There was a large difference in the incidence of delirium in the intervention group between the interim analysis (6%) and the final analysis (15%). Therefore, the intended level of power was not achieved. The reasons for this can only be speculated, as there were no changes to the study protocol or study team after the interim analysis. The missing effect of the intervention, nevertheless, is in line with the investigations from other authors. In a systematic review and meta-analysis multicomponent strategies were able to reduce the incidence of delirium in elderly patients with scheduled non-cardiac surgery. Strategies during the perioperative period (optimization of pain management or anesthesia) could only rarely improve the rate of delirium (4). However, in the special subgroup of patients in the prone position goal-directed fluid therapy improved hemodynamics and cerebral oxygenation and reduced the incidence of post-operative cognitive dysfunction (31, 32). Also, non-pharmacological multicomponent approaches were more effective. In our study pain management was sufficient in both groups and therefore no influencing factor for delirium. To this point monitoring depth of anesthesia is the best of the perioperative components to reduce delirium, especially to guide anesthetic titration during surgery and avoid long periods of burst suppression (33, 34). It must be emphasized that the evidence on this topic is still insufficient. Although delirium reduction of up to 30% was reported in the cited meta-analyses, the ENGAGES trial published in 2019 showed different results (35). Here, BIS-guided anesthesia was able to reduce the dosage of volatile anesthetics and subsequently the cumulative time with electroencephalography suppression, but not the delirium incidence within the first 5 days after major surgery. The authors attributed the differences from the meta-analyses to, among other things, older patients and compared major surgical procedures in their study. While the studies in the referred metanalyses used bispectral-index (BIS) entropy parameters in our study were comparable between groups and in the lower target range of around 40, indicating adequate depth of anesthesia.

Although in the intervention group fluid and catecholamine management was tightly controlled by the hemodynamic optimization protocol, both groups received equivalent amounts of fluids and vasopressors. This resulted in comparable intraoperative hemodynamic parameters, like sufficient mean arterial pressure and cardiac output. As cardiac output was only measured in the intervention group, this resulted in higher amounts of inotropes and therefore an increased cardiac index in this group. In the control group MAP was minimally elevated without clinical relevance. As hemodynamics did not differ between groups, not surprisingly NIRS values as surrogate parameters for cerebral perfusion and oxygenation were comparable between groups. Based on this no difference in post-operative delirium could be expected.

Although we did not see significant results in our primary endpoint, secondary analysis showed a difference in 1-year mortality between groups (*P* = 0.03). Since there was no influence on hemodynamics or cerebral oxygenation this result cannot be attributed to the intervention and we consider it as an epiphenomenon.

As a strength of our pilot study, using a screening tool for detection of patients with high risk for delirium made it possible to include a wide variety of patients with severe pre-existing conditions, extensive surgery, and an expected high risk of at least 30% for post-operative delirium. For identification of these high-risk patients, we used a modification of the risk-score published by Marcantonio (18). Several other scores have been introduced to stratify patients according to their individual risk for delirium. For example, Inouye identified five independent factors during hospitalization. However, these scores were mainly validated on general wards and the factors are not specific to surgical patients (17). In contrast, Marcantonio introduced a risk-score, that considers additional intraoperative and post-operative precipitating factors, like type of anesthesia and type of surgery. Daily post-operative visits for up to 7 days as well as inspection of the patient medical charts allowed for almost complete post-operative monitoring in order to detect all forms of delirium. The CAM-Score is the most reliable and validated score to detect delirium and is available in German. It has a sensitivity of 0.79 and a specificity of 0.97 (26).

However, there are limitations to our study: a single-center pilot trial only allows to assess the level-of-care provided in our hospital. This effect is emphasized by the already good standard hemodynamic management in the control group, that could not be further optimized by the algorithm. This lack of effect, especially in comparison with an already good control group, has already been shown, also in own work (36, 37). It might be explained by the fact that the algorithm used was based on absolute values of MAP and cardiac index. This may be a limitation of our study, as recent evidence suggests the use of individualized hemodynamic target parameters based on pre-operative measurements (38). In addition, the possibility of performance bias as reason for the missing effect of the intervention must also be considered. Above that, it must be further noted that the risk of delirium in our patient population may not have been severe enough in order for the intervention to achieve a difference.

Furthermore, hemodynamic variability must be taken into account, as it is possible that patients in the control group experienced periods of hypotension followed by periods of hypertension. However, the amount of time patients experienced intraoperative hypotension was not measured. Intervention was limited to the perioperative period as we did not provide guidelines for pre-operative optimization or therapy in the post-operative course. A further limitation is the missing information regarding post-operative inflammation as blood samples were not taken in a regular base.

Our intervention focused solely on hemodynamic optimization. In particular, no targeted intervention was provided for additional optimization of insufficient NIRS values. This is partly due to the fact that improved cardiac output was hypothesized to improve cerebral perfusion and subsequently oxygenation. Further research in this field should include management of pathological NIRS values in the algorithm, although there is only a weak association of low NIRS values and worse neurological outcome and the effect is most prominent in cardiac surgery (39, 40). An additional individualized, multi-component intervention strategy might have been more efficient in reducing delirium and should be investigated in a subsequent trial (4, 41). Lastly, the outcome-assessor was not blinded in this trial and outcome was assessed once daily, which can lead to bias. This effect might be somewhat mitigated by communicating with the team and assessing psychoactive medication utilization. However, the combination of assessment by a team member and chart review to a composite endpoint is also not ideal as it mixes different delirium screening methods. Nevertheless, because the incidences for both methods separately are comparable to the composite endpoint, the results are valid. There is a possibility that a significant amount of hypoactive delirium was missed and that the CAM-positive patients in the study had more severe delirium. Ideally, a more sensitive assessment method for delirium should be used in a follow-up trial and applied multiple times daily.

In conclusion, a goal-directed hemodynamic optimization protocol did not change hemodynamic interventions, did not improve the patients’ hemodynamics, and did not enhance cerebral oxygenation in old high-risk patients. The algorithm applied had no effect on the incidence of post-operative delirium.

## Data Availability Statement

The raw data supporting the conclusions of this article will be made available by the authors, without undue reservation.

## Ethics Statement

The studies involving human participants were reviewed and approved by the Ethikkommission der Technischen Universität München, Ismaninger Straße 22, 81675 München. The patients/participants provided their written informed consent to participate in this study.

## Author Contributions

SS was the principal investigator and developed the protocol. BU was the study statistician. SS, BJ, and KEF were involved in the ethical approval. KEF, BU, AS, BJ, MB, SJS, and SS were involved in the analysis and interpretation of the data. KEF, SS, AS, and BJ were involved in the data acquisition and quality assurance. All authors critically revised the manuscript and approved its final version.

## Conflict of Interest

BJ received honoraria for giving lectures from Pulsion Medical Systems SE (Feldkirchen, Germany). MB received research support from MSD (Haar, Germany) not related to this manuscript, received honoraria for giving lectures from GE Healthcare (Helsinki, Finland) and Grünenthal (Aachen, Germany). SJS reports grants from Reactive Robotics GmbH (Munich, Germany), grants and non-financial support from STIMIT AG (Biel, Switzerland), Liberate Medical LLC (Crestwood USA), ESICM (Geneva, Switzerland), grants, personal fees and non-financial support from Fresenius Kabi Deutschland GmbH (Bad Homburg, Germany), personal fees from Springer Verlag GmbH (Vienna, Austria) for educational purposes, non-financial support from Technical University of Munich (Munich, Germany) and from National and international societies (and their congress organizers) in the field of anesthesiology and intensive care medicine, outside the submitted work. SJS held stocks in small amounts from Rhön-Klinikum AG and holds stocks in small amounts from Alphabeth Inc., Bayer AG and Siemens AG; these holdings have not affected any decisions regarding his research or this study. The remaining authors declare that the research was conducted in the absence of any commercial or financial relationships that could be construed as a potential conflict of interest.

## Publisher’s Note

All claims expressed in this article are solely those of the authors and do not necessarily represent those of their affiliated organizations, or those of the publisher, the editors and the reviewers. Any product that may be evaluated in this article, or claim that may be made by its manufacturer, is not guaranteed or endorsed by the publisher.

## Footnotes

1
https://datatab.net
